# Until programmed death do us tolerant

**DOI:** 10.1172/JCI164858

**Published:** 2022-11-15

**Authors:** David W. Scott

**Affiliations:** Department of Medicine, Uniformed Services University of Health Sciences, Bethesda, Maryland, USA.

## Abstract

Healthy individuals are generally immunologically tolerant to proteins derived from one’s self (termed self proteins). However, patients with monogenic clotting disorders, like hemophilia A (HemA), lack central tolerance to the absent self protein. Thus, when exposed to replacement therapy, such as procoagulant factor VIII, they may mount an immune response against the very self protein that is missing. In the current issue of the *JCI*, Becker-Gotot, Meissner, et al. present data on a possible mechanism for tolerance to factor VIII in healthy individuals and the immune response in patients, involving a role of PD-1 and T regulatory cells. The findings suggest that treatment with PD-1– and PD-1L–specific reagents may induce tolerance in patients with autoimmune disease, especially those with HemA who also possess inhibiting antibodies.

## Hemophilia inhibitors

Patients with mutations in the *F8* gene, encoding coagulation protein factor VIII (FVIII), have an X-linked bleeding disorder known as hemophilia A (HemA) and lack FVIII or functional FVIII protein (1, 2). These patients may suffer recurrent spontaneous bleeds or more serious trauma-induced bleeding crises and often experience substantial morbidity due to recurrent bleeding into the joints (3, 4). Standard treatment for HemA involves infusion of recombinant FVIII or plasma-derived FVIII on demand or prophylactically. However, up to 30% of treated patients develop antibodies that can block and inhibit the function of this life-saving therapy (4, 5) since they are not immunologically tolerant to this human protein. The antibodies that neutralize FVIII function, referred to as “inhibitors” (6), pose a major clinical challenge, as once formed, inhibitor titers are difficult to reduce or eliminate.

In this issue of the *JCI*, Becker-Gotot, Meissner, and colleagues (7) examined inhibitor formation in a mouse model for HemA (HemA mice). HemA mice, which have FVIII deficiency via removal of coding exon 16, were developed by Bi et al. (8) and have been used by researchers in the field for decades. Notably, nearly 100% of these HemA mice develop inhibitors to intravenously administered FVIII, whereas approximately 30% of patients with severe HemA develop clinically relevant inhibitors. We know that the immune response to FVIII is highly T (helper) cell dependent (9), but it is regulated in FVIII-sufficient mice (and humans) by T regulatory cells (Tregs) (10).

In previous studies, Gotot and colleagues provided evidence that the regulated immune response likely requires programmed cell death protein 1 (PD-1) and programmed death ligand 1 (PD-L1) interactions (11, 12), which leads to Treg-dependent apoptosis in wild-type (WT) mice. In contrast, HemA mice lack B cell tolerance to FVIII due to defective deletion, suppression, or receptor editing from lack of exposure to FVIII epitopes during development.

Seminal studies on tolerance mechanisms took advantage of B cell receptor–transgenic (BCR-transgenic) mice (13, 14) to follow the fate of antigen-specific B cells. However, these models are not yet available for FVIII-specific BCRs. Becker-Gotot, Meissner, et al. detected FVIII-specific B cells using Alexa Fluor 647–coupled FVIII, and then examined the Alexa Fluor 647–positive cells to analyze apoptosis directly in tolerized antigen-specific B cells: a tour de force, as such cells are rare in nontransgenic mice, occurring with a frequency of approximately 1/1000–1/2000). The flow cytometry approach was also challenging because FVIII is a relatively sticky protein with the ability to bind to other (e.g., endothelial) cell surfaces, so the specificity of FVIII binding to specific B cells is critical and this analysis needs to be validated, e.g., by competition with unlabeled FVIII or blocking with anti-IgM. Nonetheless, the authors found that the number of FVIII-specific B cells in naive mice (WT or HemA) was far lower than in immunized mice. A well-established principle is that tolerance to FVIII or other self-antigens in healthy mice is not broken in the absence of added adjuvants. In contrast, KO mice are expected to respond as we would to most antigens that we lack. Importantly, FVIII is very immunogenic even when given intravenously, a usually tolerogenic route. Whether this immunogenicity has to do with thrombin generation has been debated (15, 16).

## Role of the PD-1/PD-L1 pathway in immune regulation

PD-L1 was first described by Honjo’s group in 1992 (17) and extensively studied by the Sharpe and Freeman groups at Harvard (11). PD-1 (also known as CD279) is an activation-induced member of the CD28/CTLA-4 family, known for suppressing conventional T cell responses (18), and allowing tumors to escape surveillance (19). Thus, PD-1 (or similar) blocking antibodies have emerged with potential as anticancer drugs. Interestingly, patients treated with such PD-1 blocking antibodies often develop autoantibodies, a result suggesting that PD-1 has a critical role in immune regulation and tolerance (12).

**Figure 1:**
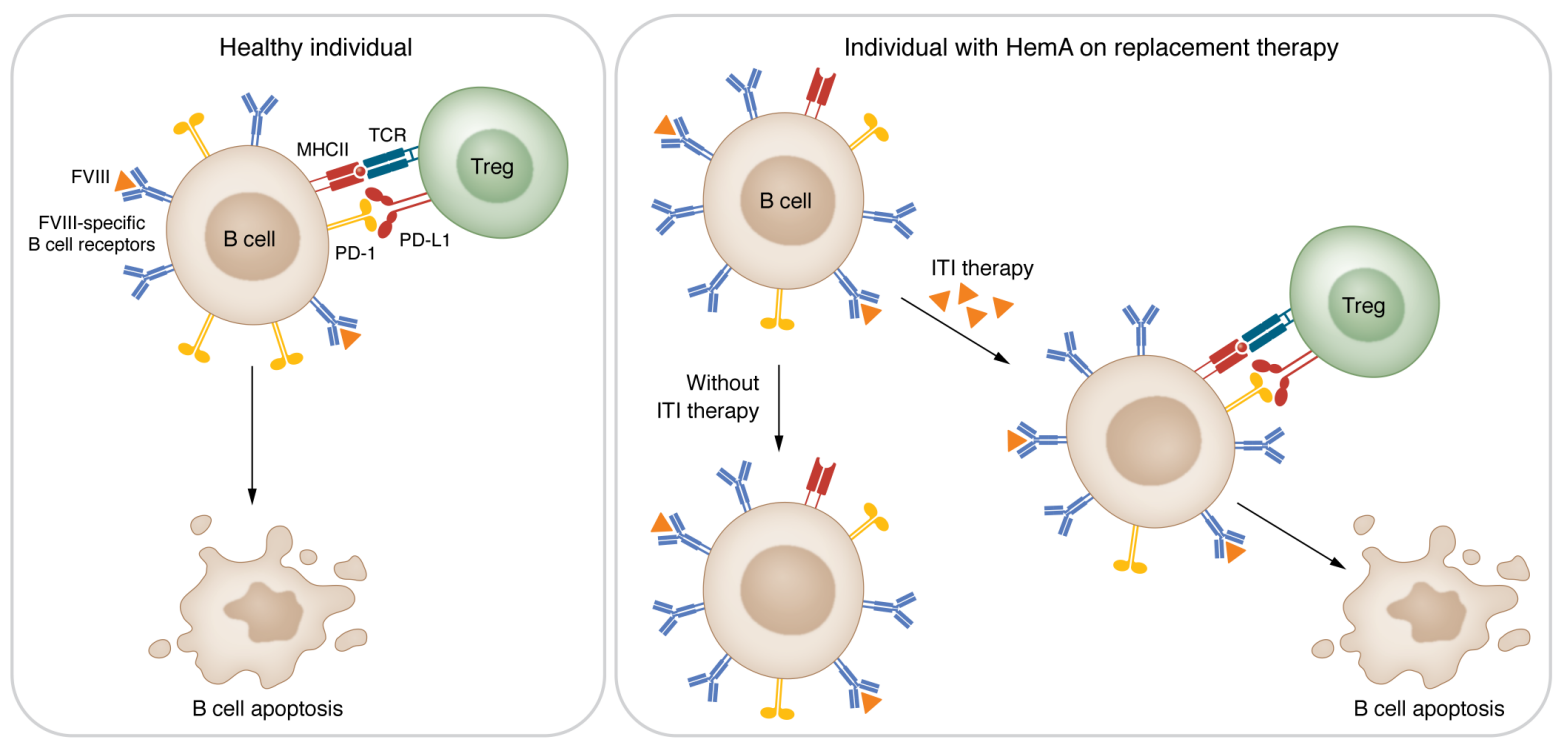
A model for PD-1/PD-L1 pathway–driven immune tolerance. The interaction of PD-L1 on Treg cells with PD-1 on antigen-specific B cells mediates tolerance via apoptosis, resulting in decreased numbers of B cells that are specific for self-antigens in healthy individuals. B cells from individuals with hemophilia A (HemA) can be stimulated to produce antibodies against FVIII. Repetitive injections of FVIII induces immune tolerance via a PD-1/PD-L1–mediated process in patients with HemA who have inhibitors.

PD-1 expression on FVIII-specific B cells in immunized HemA mice was lower than on those remaining antigen-specific B cells in WT animals, whereas annexin V–positive (indicating apoptotic) B cells were more evident in WT mice. This result suggested to the authors that PD-L1^+^ Tregs that recognize specific B cells that present FVIII peptides via MHC II were responsible for potentially deleting FVIII-specific B cells during developmental exposure to this self-antigen. Indeed, when Becker-Gotot, Meissner, et al. treated WT mice with an antibody that blocks PD-1 signaling, FVIII-specific B cells were rescued from deletion, allowing the cells to expand and produce anti-FVIII antibody. Moreover, a PD-1–stimulating antibody treatment in HemA mice led to suppression of anti-FVIII responsiveness. While the authors concede that other checkpoint inhibitors may be involved, their data support an import role of the PD-1/PD-L1 pathway and Treg-driven apoptosis in this model of tolerance (Figure 1).

For decades, efforts to reduce or eradicate inhibitor formation, i.e., to induce tolerance, have included treatment with repeated and high doses of FVIII (20). The protocol for immune tolerance induction (ITI) (21) to FVIII is costly, time consuming, and challenging for patients and their families. Moreover, ITI only succeeds clinically in approximately 70% of patients. Becker-Gotot, Meissner, et al. further explored the relevance of the ITI model to HemA therapy in two ways. First, they attempted a model of ITI in mice with repeated injections of FVIII in immunized HemA mice, leading to some diminution of inhibitor titers. This ITI-like protocol caused an expansion of PD-L1–expressing FVIII-specific-tetramer–positive induced Tregs in HemA mice, thus further implying a role of Tregs in self-tolerance to FVIII. Secondly, in a tour de force effort, they examined FVIII-binding B cells in both healthy adults and in one HemA patient who possessed inhibitors. It was necessary to use adults to obtain sufficient blood for analysis due to the rare frequency of such cells and difficulty in obtaining sufficient blood from young patients. Similar to results in mice, healthy donors had high levels of PD-1 mRNA in their FVIII-specific B cells, while PD-1 mRNA was not detectable in the FVIII-specific B cells of the HemA patient prior to ITI initiation. A few days after ITI was started, both PD-1 and Fas were undetectable in the patient’s FVIII-specific B cells. These results are anecdotal (*n* = 1), but suggestive of the results obtained in HemA mice. Further studies in a small cohort of patients suggested that ITI may establish PD-1–mediated immune tolerance and FVIII-specific Tregs in HemA patients.

Given that we can extrapolate from these data, the combination of both the murine studies in HemA and WT mice and preliminary results with HemA patients support the notion of a critical role for PD-1–mediated suppression as an important mechanism for tolerance.

## ITI for inhibitors

ITI is an expensive and time-consuming process and, as noted, not universally successful. Moreover, the advent and increased usage of the bispecific antibody emicizumab (22), which bypasses FVIII to create a tenase complex, has led to a hot debate among hemophilia treaters about whether ITI should be attempted. But one should be aware that the maintenance of tolerance still requires the antigen (23), FVIII, since new B cells are continually being generated from bone marrow precursors. Hence, a hybrid model using emicizumab and low-dose FVIII treatment needs to be considered for patients who choose treatments with emicizumab (or other potential non-FVIII treatments to achieve hemostasis). Still, the results of Becker-Gotot, Meissner, et al. remind us that tolerance is an active process and that understanding and application of PD-1– and PD-1L–specific reagents may be useful to induce tolerance clinically, not only in the presence of hemophilia inhibitors but also in autoimmune diseases or other adverse immune responses.

## Version 1 11/15/2022

Electronic publication
